# CCAL1 enhances osteoarthritis through the NF‐κB/AMPK signaling pathway

**DOI:** 10.1002/2211-5463.12989

**Published:** 2020-10-24

**Authors:** Hanzhong Zhu, Hongyu Yan, Junan Ma, Hua Zhang, Jidong Zhang, Zhiheng Hu, Yunliang Guo

**Affiliations:** ^1^ Department of Orthopaedic Surgery Chengwu People's Hospital Heze China; ^2^ Department of Medical College Qingdao University Qingdao China

**Keywords:** CCAL1, chondrocytes, fibrosis, NF‐κB/AMPK signaling pathway, osteoarthritis, proliferation

## Abstract

Osteoarthritis (OA) is a chronic joint disease characterized by articular cartilage degeneration and secondary osteogenesis. It has been previously demonstrated that the CCAL1 locus is the gene encoding tumor necrosis factor receptor superfamily member 11B (*TNFRSF11B*). The purpose of this study was to demonstrate the role of CCAL1 in OA progression and to elucidate its molecular mechanisms. We report that CCAL1 is highly expressed in the cartilage of OA patients and its expression level is positively correlated with the severity of OA. We found that CCAL1 causes a switch to the fibrosis‐prone phenotype of Human Chondrocyte‐Osteoarthritis (HC‐OA) cells. In addition, CCAL1 enhances cell viability and promotes the proliferation of HC‐OA cells. Finally, the detection of proteins associated with the NF‐κB/AMPK signaling pathway by western blot suggested that CCAL1 exerts its role on HC‐OA cells by activating the NF‐κB signaling pathway and inhibiting the AMPK signaling pathway, which was verified through the addition of NF‐κB inhibitor caffeic acid phenethyl ester (CAPE) and AMPK activator 5‐aminoimidazole‐4‐carboxamide riboside (AICAR). In summary, we report that CCAL1 may promote OA through the NF‐κB and AMPK signaling pathways.

## Graphical abstract

CCAL1, a member of the tumor necrosis factor receptor superfamily 11B, encodes osteoprotegerin. We report that CCAL1 is highly expressed in osteoarthritis (OA) tissues and Human Chondrocyte‐Osteoarthritis (HC‐OA) cells and its expression level is positively correlated with the severity of OA. CCAL1 could activate NF‐κB and inhibit AMPK pathways to promote HC‐OA cell fibrosis, viability and proliferation.

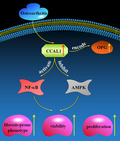

**Abbreviations**
AICAR: 5‐aminoimidazole‐4‐carboxamide riboside
AMPK: adenosine monophosphate‐activated protein kinase
CAPE: caffeic acid phenethyl ester
HC‐OA: human chondrocyte‐osteoarthritis
OA: osteoarthritis
OD: optical density
OPG: osteoprotegerin
PVDF: polyvinylidene difluoride
SD: standard deviation
TNFRSF11B: tumor necrosis factor receptor superfamily member 11B

Osteoarthritis (OA) is a chronic degenerative joint disease, and its incidence increases with age [1]. OA is the main cause of joint synovial membrane, cartilage, and subchondral bone lesions, and even leading to joint structural deformities and dysfunction [2]. OA has posed a threat to the life quality of patients and effective clinical treatment is urgently needed. Recent studies have demonstrated the identity of CCAL1 as *TNFRSF11B*, which encodes the osteoprotegerin (OPG) [3]. The study of Pilichou *et al*. has demonstrated that the level of OPG in the synovial fluid of OA patients is closely related to the severity of knee OA. With the severity of knee osteoarthritis, the expression level of OPG increased gradually [4]. Ramos *et al*. have demonstrated that the upregulation of *TNFRSF11B* in OA patients’ cartilage is a general phenomenon in the pathophysiological process of OA [5].

Besides, adenosine monophosphate‐activated protein kinase (AMPK) is reported to improve the anti‐stress ability and viability of chondrocytes by regulating the activity of its downstream target molecules in articular cartilage [6]. AMPK signaling plays a regulatory role in the chondrocyte apoptosis and cartilage degeneration of OA rat models [7]. In addition, lots of studies have suggested that the occurrence and development of osteoarthritis are related to the excessive activation of NF‐κB [8]. NF‐κB signaling pathway is implicated in the proliferation and apoptosis of OA chondrocytes [9] and has been suggested as the therapeutic target for OA [10]. However, whether CCAL1 can function on OA chondrocytes via the NF‐κB/AMPK signaling pathway has not been solved. Therefore, the purpose of this study is to investigate the potential roles and mechanisms of CCAL1 in OA chondrocytes and to provide a potential therapeutic target for the treatment of OA.

## Materials and methods

### Sample collection

A total of 68 OA patients aged 55–64 years admitted in the orthopedic surgical of Chengwu county people's hospital, were included in our study. The pathological cartilage was acquired during total hip replacement from end‐stage symptomatic hip OA patients and graded according to Outerbridge classification. A total of 14 patients were diagnosed as Grade I, 18 as Grade II, 20 as Grade III and 16 as Grade IV. The control cartilage samples were collected from 20 patients without joint disease under total hip replacement. The study methodologies conformed to the standards set by the declaration of Helsinki. All the samples came from patients without autoimmune, gouty, and infectious arthritis. All the patients or their guardians had signed the informed written consent before the study. This study was approved by the Ethics Committee of Chengwu people's Hospital.

### Cells culture

Human articular chondrocyte C‐20/A4 cells and HC‐OA cells from OA patients were purchased from ATCC (Manassas, VA, USA) and Shanghai yagiBio Co. Ltd. (Shanghai, China), respectively. C‐20/A4 cell line is a widely available chondrocyte model. HC‐OA was derived from human articular cartilage from OA donor, which provided a useful model for studying the biological changes of chondrocytes in the abnormal joint environment. C‐20/A4 cells and HC‐OA cells were extracted from the liquid nitrogen and resuscitated separately. C‐20/A4 cells were maintained in DMEM/F12 high‐glucose medium (containing 10% FBS, 100 U·mL^‐1^ penicillin, 50 U/mL streptomycin, 50 mg·L^−1^ ascorbic acid and 300 mg·L^−1^ l‐glutamate) at 37 °C with 5% CO_2_. HC‐OA cells were cultured in α‐MEM medium containing 10% FBS at 37 °C with 5% CO_2_.

### Cell transfection

The CCAL1 gene fragment was synthesized and inserted into LV5 lentiviral vector. The LV5‐CCAL1 recombinant plasmid and empty LV5 vector were transferred into HC‐OA cells separately by using 100 nm diluted Lipofectamine 2000 (Invitrogen, Grand Island, NY, USA). After transfection for 48 h, the cultures were centrifugated and the cell suspension was collected. Subsequently, HC‐OA cells (1 × 10^5^ cells per well) were seeded in 24‐well plate, added with 6 μg·mL^−1^ polybrene and moderate cell suspension. After incubated at 37 °C for 48 h, the positively transfected HC‐OA cells were selected for further analysis. Meanwhile, the sequence of inhibitor NC was 5'‐CAGUACUUUUGUGUAGUACAA‐3' and the CCAL1 inhibitor was 5'‐CAGGCACUUGAGGCUUUCAGUGAUA‐3'. Lipofectamine 2000 (Invitrogen) was used for cell transfection according to the manufacturer's protocol.

### Western blot analysis

The total protein of transfected cells was extracted by RIPA lysis buffer (Beyotime, Shanghai, China). The protein quality was evaluated by the BCA protein assay kit (Thermo Fisher Scientific, Waltham, MA, USA). After purified, the protein was separated by 12% SDS‐PAGE and then transferred to the polyvinylidene difluoride (PVDF) membrane (Millipore, Bedford, MA, USA). The PVDF membranes were blocked by 5% (w/v) fat‐free milk for 1 h, followed by incubation overnight at 4 °C with corresponding primary antibodies including CCAL1 (1 : 1000), Collagen I (1 : 1000), SOX9 (1 : 1000), Collagen II (1 : 1000), IκB (1 : 1000), p65 (1 : 1000), AMPK (1 : 1000), p‐AMPK (1 : 1000), β‐actin (1 : 1000). After washed with PBS, membranes were incubated with corresponding secondary antibodies at 37 °C for 1 h. Finally, the ECL detection kit (Beyotime, China) was used to detect the results.

### qRT–PCR analysis

Total RNA was extracted using TRIzol reagent (Invitrogen). Reverse transcription reaction was performed by Takara RNA PCR Kit (Takara, Kyoto, Japan). Real‐time quantitative PCR was executed by SYBR Green detection system (Takara). The primer sequences were listed below: CCAL1 forward: 5′‐ACCCAGAAACTGGTCATCAGC‐3′, CCAL1 reverse: 5′‐CTGCAATACACACACTCATCACT‐3′, Collagen I forward: 5′‐CGGTGGTTACGACTTTGGTT‐3′, Collagen I reverse: 5′‐TCAGAGTGGCATCGACTTCA‐3′, SOX9 forward: 5′‐AGCTCACCAGACCCTGAGAA‐3′, SOX9 reverse: 5′‐GATTCTCCAATCGTCCTCCA‐3′, Collagen II forward: 5′‐AACACTGCCAACGTCCAGAT‐3′, Collagen II reverse: 5′‐CTGCAGCACGGTATAGGTGA‐3′, β‐actin forward: 5′‐GGCCCAGAGCAAGAGAGGTATCC‐3′, β‐actin reverse: 5′‐ACGCACGATTTCCCTCTCAGC‐3′. The 2^‐ΔΔCt^ method was used to calculate the relative expression of transcripts.

### Colony formation assay

Briefly, HC‐OA cells were digested with trypsin, seeded into 6‐well plates with a density of 500 cells/well, and cultured in DMEM containing 10% FBS. The medium was refreshed every 3 days. After 2 weeks, the cells were fixed with 4% paraformaldehyde for 15 min and stained using 1% crystal violet. The number of cell colonies was counted and photographed.

### CCK‐8 assay

Briefly, cells were seeded into a 96‐well plate and cultured for 0, 12, 24, 48, 72 h respectively. Then, the reaction systems of 10 μL CCK‐8 and 100 μL PBS were added to each well and cells were incubated at room temperature for 2 h. Finally, the optical density (OD) values at 450 nm were detected by Thermo Fisher Scientific Multiskan FC.

### Statistics analysis

Statistical analysis was performed using spss 20.0 software (SPSS Inc, Chicago, IL, USA). Data were presented as means ± standard deviation (SD). Differences between groups were analyzed by Student′s *t*‐test or one‐way ANOVA. Each experiment was performed in three replications. *P* < 0.05 and *P* < 0.01 represented differences and significant differences, respectively.

## Results

### CCAL1 was highly expressed in patients with OA

**Fig. 1:**
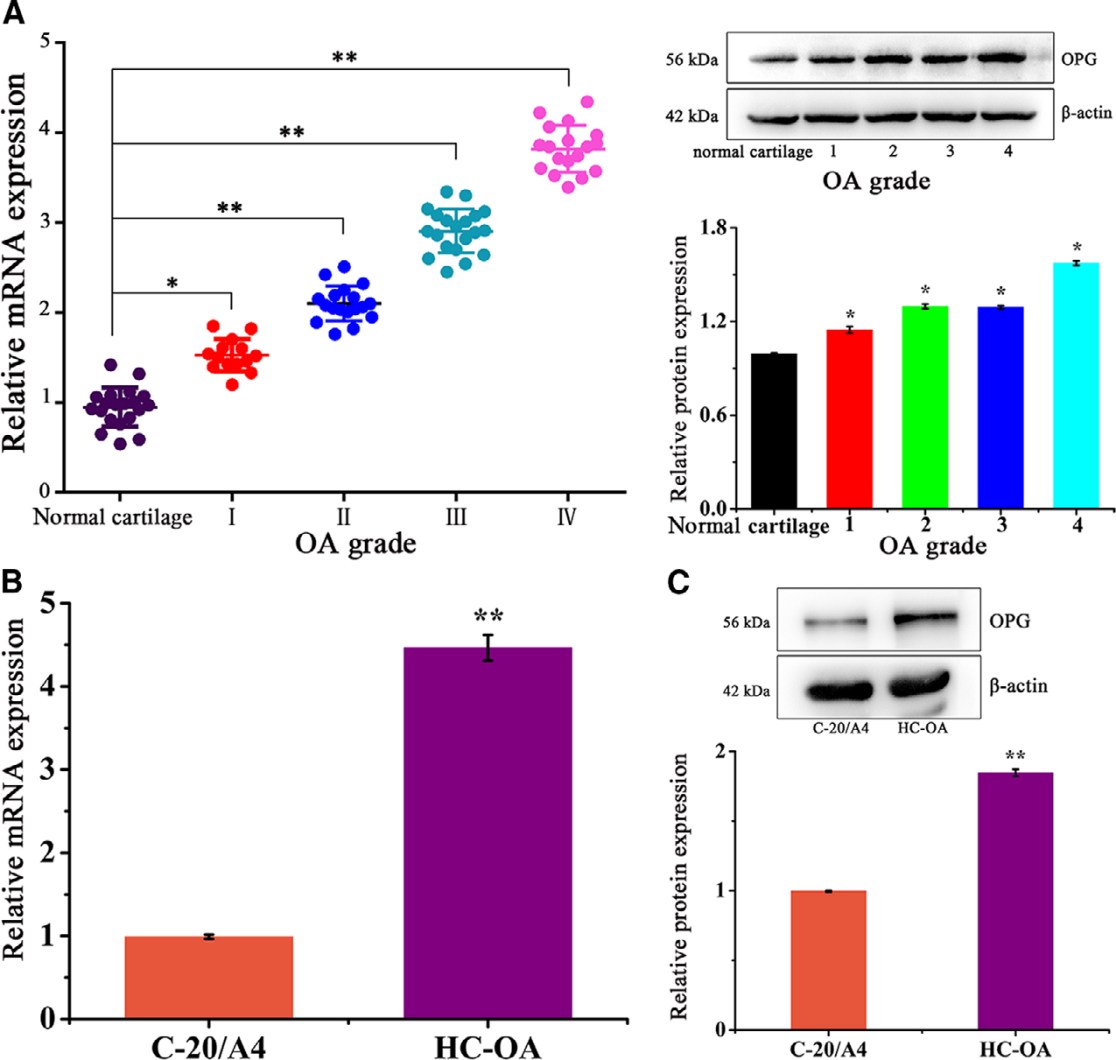
CCAL1 is overexpressed in patients with OA. (A) qRT–PCR and WB were performed to examine CCAL1 expression in 68 pairs of OA tissues and control OA tissues. (B) qRT**–**qPCR was performed to examine CCAL1 mRNA expression in C‐20/A4 cells and HC‐OA cells. (C) WB was performed to examine the intracellular CCAL1 protein expression level in C‐20/A4 cells and HC‐OA cells. Data were analysed using Student’s t‐test and are expressed as the mean ± SD of three experiments. Symbols represent statistical significance (*, *P* < 0.05; **, *P* < 0.01).

The expression levels of CCAL1 in cartilage tissues of patients with OA at different clinical stages and subjects without joint disease were detected by qRT**–**PCR and WB analysis. The results suggested that CCAL1 expression was significantly higher in OA patients compared with control (*P* < 0.01). The expression of CCAL1 was increased in OA patients in a grade‐dependent manner (Fig. 1A). Besides, the expression levels of CCAL1 in C‐20/A4 cells and HC‐OA cells were also detected by qRT**–**PCR and WB analysis. The expression of CCAL1 in HC‐OA cells was remarkably higher than that of C‐20/A4 at both mRNA and protein levels (*P* < 0.01, Fig. 1B,C). The above results confirmed that CCAL1 was overexpressed in osteoarthritis patients and HC‐OA cells, and the expression level was positively correlated with the severity of OA.

### CCAL1 overexpression promoted fibrosis, cell viability and proliferation of HC‐OA cells

**Fig. 2:**
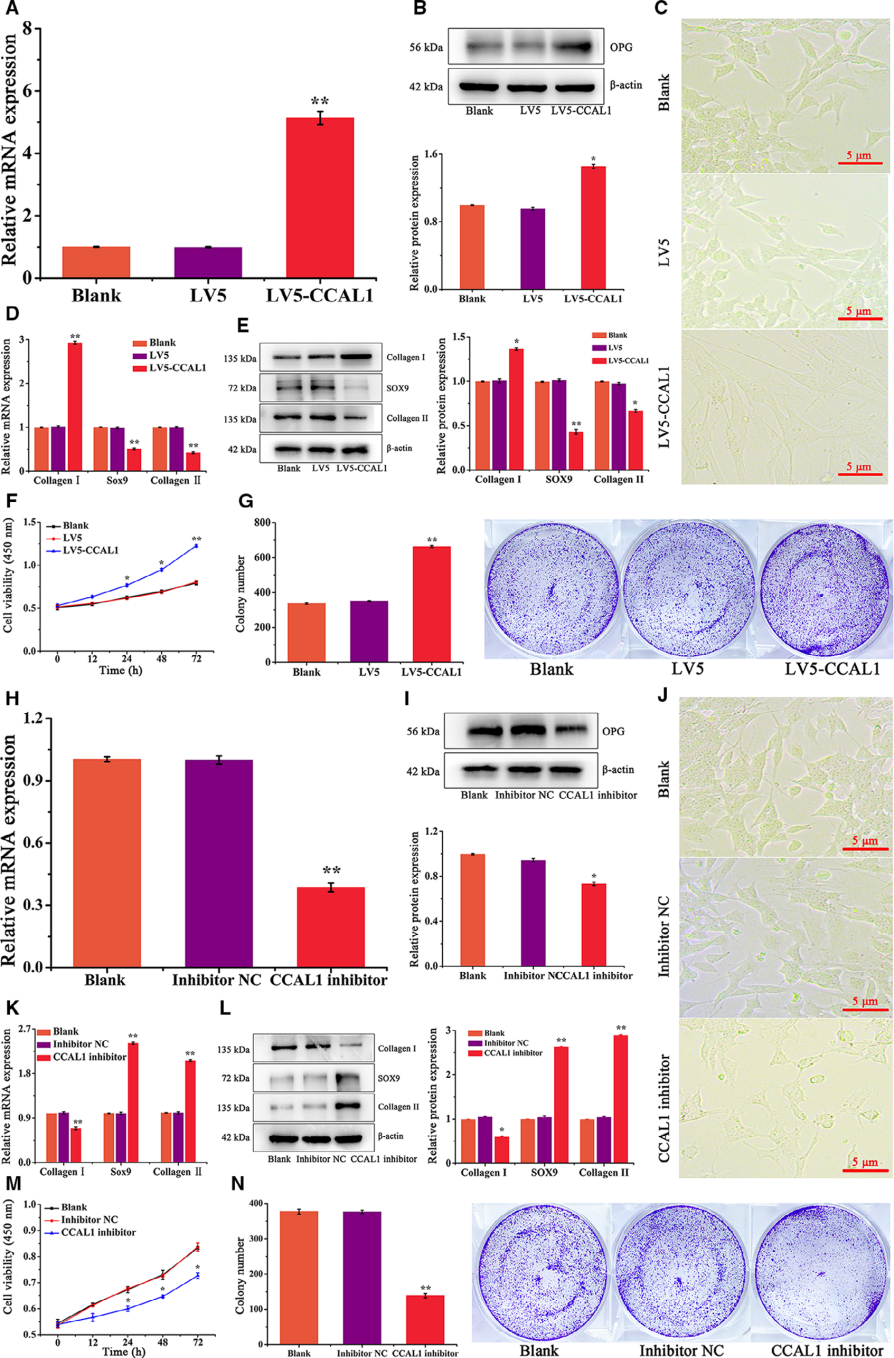
The effect of CCAL1 on fibrosis and proliferation of HC‐OA cells. (A) qRT**–**PCR was performed to analyze the expression of CCAL1 mRNA in HC‐OA cells after different treatments (Blank, LV5, LV5‐CCAL1). (B) WB was performed to analyze the expression of the intracellular CCAL1 protein in HC‐OA cells after different treatments (Blank, LV5, LV5‐CCAL1). (C) HC‐OA cells were observed after different treatments (Blank, LV5, LV5‐CCAL1). Scale bars, 5 μm (*n* = 3). (D) qRT**–**PCR was performed to analyze the expression of Collagen Ⅰ, Sox9, Collagen Ⅱ mRNAs in HC‐OA cells after different treatments (Blank, LV5, LV5‐CCAL1). (E) WB was performed to analyze the expression of Collagen Ⅰ, Sox9, Collagen Ⅱ proteins in HC‐OA cells after different treatments (Blank, LV5, LV5‐CCAL1). (F) The cell viability of HC‐OA cells after different treatments (Blank, LV5, LV5‐CCAL1) was detected by CCK‐8 assay. (G) The proliferation capacity of HC‐OA cells after different treatments (Blank, LV5, LV5‐CCAL1) was detected by clone formation assay. (H) qRT**–**PCR was performed to analyze the expression of CCAL1 mRNA in HC‐OA cells after different treatments (Blank, inhibitor NC and CCAL1 inhibitor). (I) WB was performed to analyze the expression of the intracellular CCAL1 protein in HC‐OA cells after different treatments (Blank, inhibitor NC and CCAL1 inhibitor). (J) HC‐OA cells were observed after different treatments (Blank, inhibitor NC and CCAL1 inhibitor). Scale bars, 5 μm (*n* = 3). (K) qRT**–**PCR was performed to analyze the expression of Collagen Ⅰ, Sox9, Collagen Ⅱ mRNAs in HC‐OA cells after different treatments (Blank, inhibitor NC and CCAL1 inhibitor). (L) WB was performed to analyze the expression of Collagen Ⅰ, Sox9, Collagen Ⅱ proteins in HC‐OA cells after different treatments (Blank, inhibitor NC and CCAL1 inhibitor). (M) The cell viability of HC‐OA cells after different treatments (Blank, inhibitor NC and CCAL1 inhibitor) were detected by CCK‐8 assay. (N) The proliferation capacity of HC‐OA cells after different treatments (Blank, inhibitor NC and CCAL1 inhibitor) were detected by clone formation assay. Data were analysed using Student’s t‐test and are expressed as the mean ± SD of three experiments. Symbols represent statistical significance (*, *P* < 0.05; **, *P* < 0.01).

To further investigate the role of CCAL1 overexpression on HC‐OA cells, HC‐OA cells were transfected with LV5‐CCAL1 and empty vector LV5. The transfection efficiency was verified by qRT**–**PCR and WB analysis. The expression of CCAL1 at mRNA level was significantly increased in LV5‐CCAL1 group, compared with the Blank and LV5 group (*P* < 0.01, Fig. 2A). Meanwhile, the protein expression of CCAL1 in the LV5‐CCAL1 group was the highest among groups (*P* < 0.05), and there was no significant difference in the expression of CCAL1 between Blank and LV5 group (Fig. 2B), which agreed with the results of qRT**–**PCR analysis and demonstrated the successful transfection of CCAL1 in HC‐OA cells. Next, by observation of cell phenotype, we found that HC‐OA cells in the LV5‐CCAL1 group showed fibrosis‐prone phenotype compared with the other two groups (Fig. 2C). Subsequently, qRT**–**PCR was used to verify the expression levels of fibrosis‐related genes. Compared with the blank group and LV5 group, the expression levels of Sox9 and Collagen II were significantly decreased in the LV5‐CCAL1 group, while the expression level of Collagen I was remarkably elevated (*P* < 0.01, Fig. 2D). Then, we detected the expression of proteins related to cell fibrosis by WB and found that compared with the Blank and LV5 groups, the expression level of Collagen I in the LV5‐CCAL1 group was increased, and the expression levels of Sox9 and Collagen II were reduced (Fig. 2E). The results suggested that CCAL1 plays an important role in the regulation of chondrocyte fibrosis in OA.

To further investigate the effects of CCAL1 on the viability and proliferation of HC‐OA cells, we performed CCK‐8 and colony formation assays. Through the results of CCK‐8 assay, we found that the cell viability in the LV5‐CCAL1 group was significantly increased compared with the other two groups (Fig. 2F). The results of colony formation assay demonstrated that the number of colonies in the LV5‐CCAL1 group increased significantly compared with Blank and LV5 groups. The number of colonies in the LV5‐CCAL1 group (662 ± 5.51) was significantly higher than the Blank (351 ± 2.01) and LV5 group (337 ± 4.36) (*P* < 0.01, Fig. 2G). The results indicated that CCAL1 could increase the viability and proliferation of HC‐OA cells.

### Inhibition of CCAL1 expression decreased fibrosis, cell viability and proliferation of HC‐OA cells

The effect of CCAL1 knockdown on the fibrosis, cell viability and proliferation of HC‐OA cells was evaluated. HC‐OA cells were transfected with inhibitor NC and CCAL1 inhibitor, respectively. Then the transfection efficiency of CCAL1 was verified by qRT**–**PCR and WB analysis. Results showed that the expression of CCAL1 at mRNA and protein level was declined in the CCAL1 inhibitor group, compared with the Blank and inhibitor NC group (Fig. 2H,I), indicating that CCAL 1 inhibitor transfection was successful.

As shown in Fig. 2J, the switching of the fibrosis‐prone phenotype was attenuated in the cells after knockdown of CCAL1. Then, the expression of cell fibrosis‐related genes was detected. From the results of qRT**–**PCR analysis, we found that the expression level of Collagen I in the CCAL1 inhibitor group was significantly reduced, while the expression levels of Sox9 and Collagen II were significantly increased (*P* < 0.01, Fig. 2K). At the same time, the protein expression levels of Collagen I was decreased in HC‐OA cells after transfected with CCAL1 inhibitor, while the expression levels of Collagen II and Sox9 were significantly increased (Fig. 2L).

Finally, the effects of CCAL1 on the cell viability and proliferation of HC‐OA cells were verified by CCK‐8 and cloning formation assays. As shown in Fig. 2M, the cell viability in the CCAL1 inhibitor group was significantly reduced compared with the other two groups (*P* < 0.05, Fig. 2M). The colony formation assay showed that compared with the Blank group (377 ± 7.02) and inhibitor NC group (376 ± 5.13), the number of colonies in the CCAL1 inhibitor group (138 ± 6.65) was significantly reduced (*P* < 0.01, Fig. 2N). The results above indicated that inhibition of CCAL1 expression could relieve fibrosis and proliferation of HC‐OA cells.

### CCAL1 activated the NF‐κB signaling pathway

**Fig. 3:**
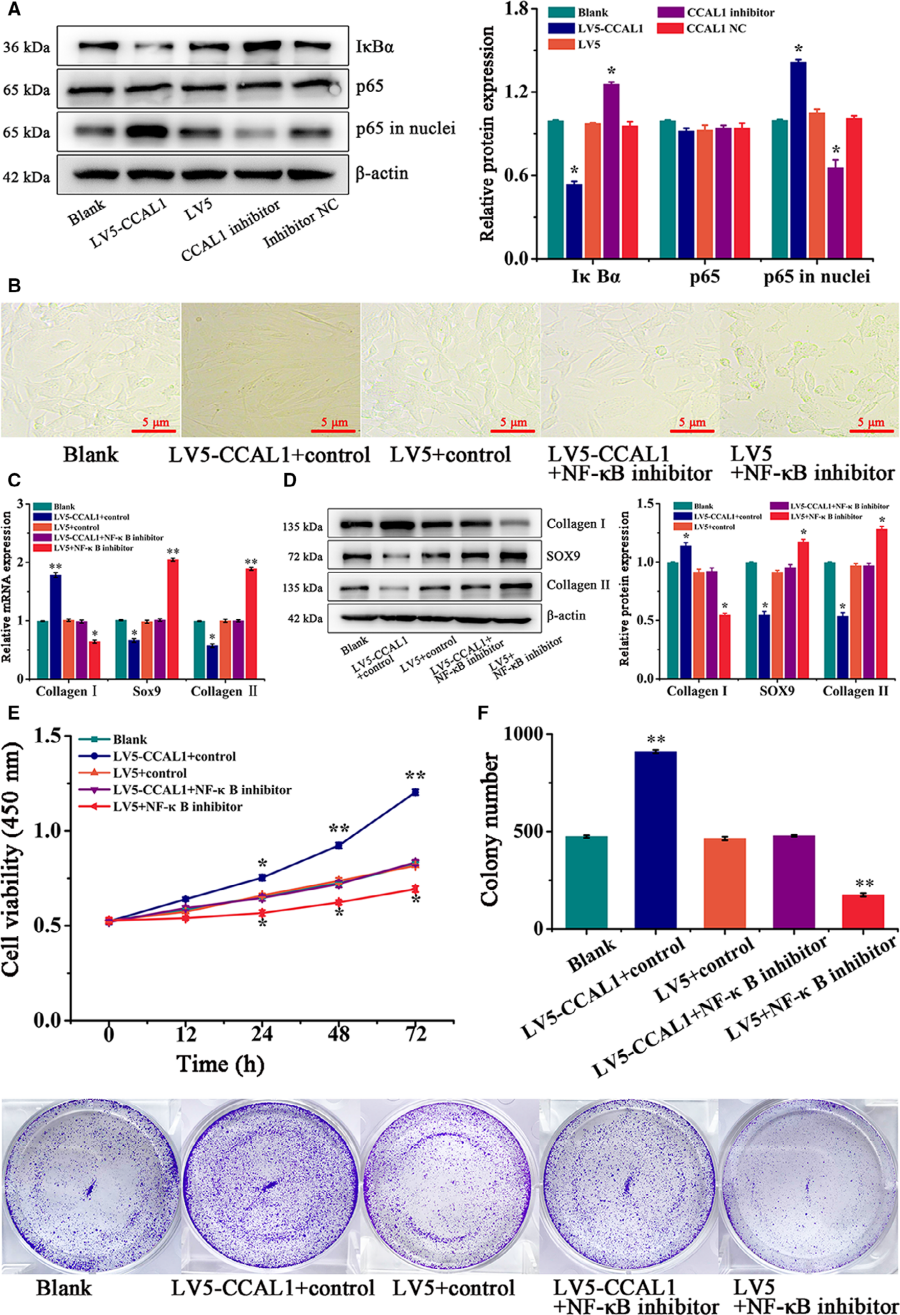
CCAL1 activates NF‐κB signaling pathway. (A) WB was performed to analyze the expression of IκBα, p65 and p65 in nuclei proteins in HC‐OA cells after different treatments (Blank, LV5‐CCAL1, LV5, CCAL1 inhibitor, inhibitor NC). (B) HC‐OA cells were observed after different treatments (Blank, LV5‐CCAL1 + control, LV5 + control, LV5‐CCAL1 + NF‐κB inhibitor, LV5 + NF‐κB inhibitor). Scale bars, 5 μm (*n* = 3). (C) qRT**–**PCR was performed to analyze the expression of Collagen Ⅰ, Sox9 and Collagen Ⅱ mRNAs in HC‐OA cells after different treatments (Blank, LV5‐CCAL1 + control, LV5 + control, LV5‐CCAL1 + NF‐κB inhibitor, LV5 + NF‐κB inhibitor). (D) WB was performed to analyze the expression of Collagen Ⅰ, Sox9, Collagen Ⅱ proteins in HC‐OA cells after different treatments (Blank, LV5‐CCAL1 + control, LV5 + control, LV5‐CCAL1 + NF‐κB inhibitor, LV5 + NF‐κB inhibitor). E. The cell viability of HC‐OA cells after different treatments (Blank, LV5‐CCAL1 + control, LV5 + control, LV5‐CCAL1 + NF‐κB inhibitor, LV5 + NF‐κB inhibitor) were detected by CCK‐8 assay. F. The proliferation capacity of HC‐OA cells after different treatments (Blank, LV5‐CCAL1 + control, LV5 + control, LV5‐CCAL1 + NF‐κB inhibitor, LV5 + NF‐κB inhibitor) were detected by clone formation assay. Data were analysed using Student’s t‐test and are expressed as the mean ± SD of three experiments. Symbols represent statistical significance (*, *P* < 0.05; **, *P* < 0.01).

In order to verify whether CCAL1 promotes fibrosis and proliferation of HC‐OA cells by the NF‐κB signaling pathway, the expression of NF‐κB signaling pathway‐related core factors were investigated after CCAL1 overexpression or inhibition. Results showed that the expression level of IκBα was significantly declined in LV5‐CCAL1 group, while remarkably elevated in CCAL1 inhibitor group (*P* < 0.05). By contrast, the p65 in nuclei expression level was significantly increased in the LV5‐CCAL1 group and declined in the CCAL1 inhibitor group (*P* < 0.05, Fig. 3A).

To further determine the effect of CCAL1 on the switching of fibrosis‐prone phenotype and proliferation by activating the NF‐κB signaling pathway, we added NF‐κB signaling pathway inhibitors into HC‐OA cells. By observation of cell phenotype via microscopy, we found that the trend of fibrosis‐prone phenotype switching of the cells was significantly improved in the LV5‐CCAL1 + inhibitor control group compared with the Blank group, LV5 + inhibitor control group and LV5‐CCAL1 + NF‐κB inhibitor group, while in LV5 + NF‐κB inhibitor group this trend was significantly reduced (Fig. 3B). After that, the expression levels of fibrosis‐related genes were detected by qRT**–**PCR and WB analysis. The results showed that compared with the other three groups, the expression of Collagen I levels significantly increased, while the expression of SOX and Collagen II were significantly reduced in LV5‐CCAL1 + inhibitor control group, the expression level of Collagen I in LV5 + NF‐κB inhibitor group was significantly reduced, while the expression levels of Sox9 and Collagen II were significantly increased (Fig. 3C,D). CCK‐8 assay showed that the cell viability was significantly increased in the LV5‐CCAL1 + inhibitor control group and decreased in the LV5 + NF‐κB inhibitor group, compared with the Blank group, LV5 + inhibitor control group and LV5‐CCAL1 + NF‐κB inhibitor group (Fig. 3E). Moreover, the number of colonies increased significantly in LV5‐CCAL1 + inhibitor control group (910 ± 8.62) and decreased significantly in LV5 + NF‐κB inhibitor group (175 ± 8.54) compared with Blank group (474 ± 6.43), LV5 + inhibitor control group (464 ± 9.02) and LV5‐CCAL1 + NF‐κB inhibitor group (464 ± 9.02) (*P* < 0.01, Fig. 3F). Thus, we speculated that CCAL1 could promote fibrosis, cell viability and proliferation of HC‐OA cells by activating the NF‐κB signaling pathway.

### CCAL1 inactivated the AMPK signaling pathway

**Fig. 4:**
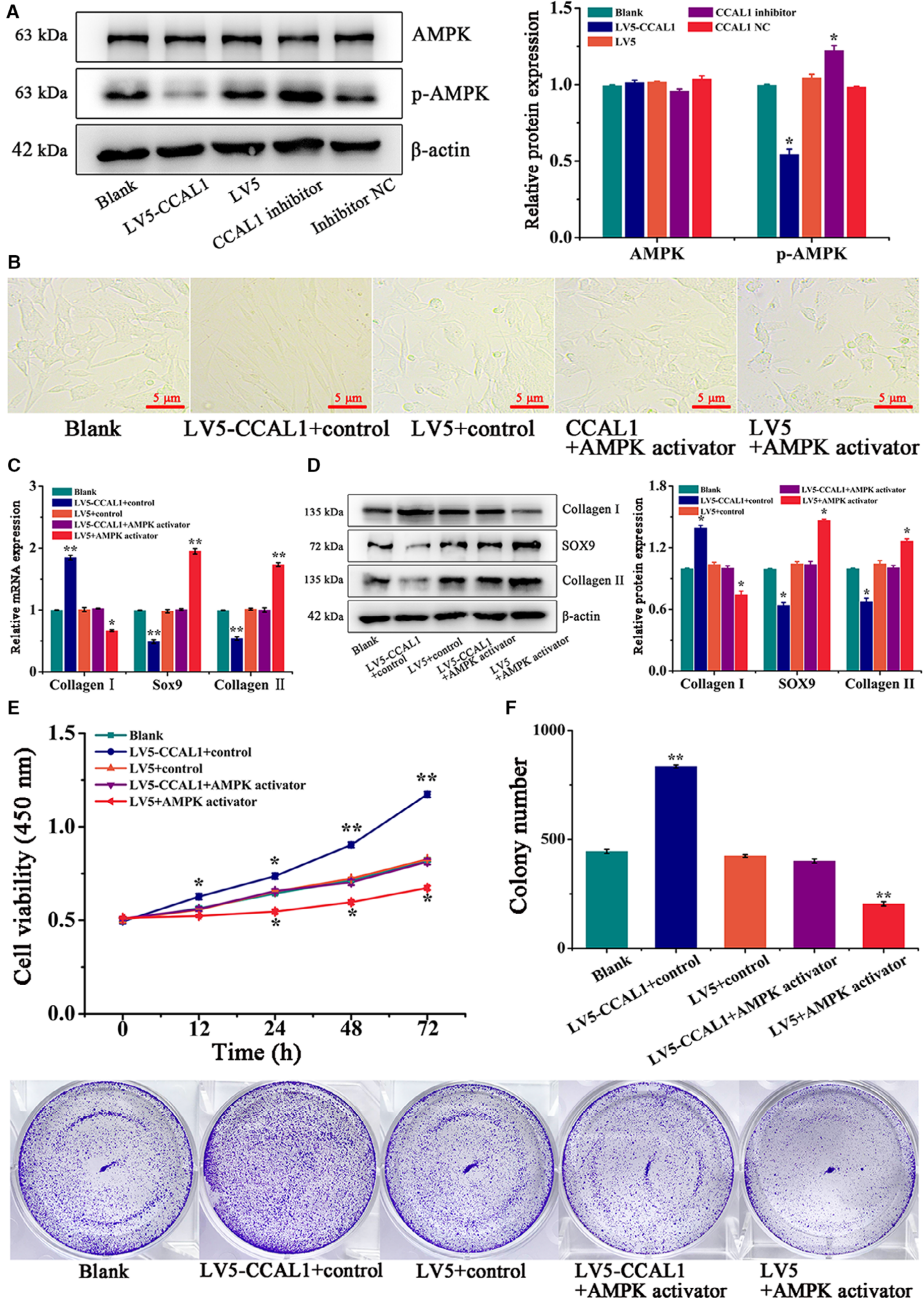
CCAL1 inactivates AMPK signaling pathway. (A) WB was performed to analyze the expression of AMPK and p‐AMPK proteins in HC‐OA cells after different treatments (Blank, LV5‐CCAL1, LV5, CCAL1 inhibitor, inhibitor NC). (B) HC‐OA cells were observed after different treatments (Blank, LV5‐CCAL1 + control, LV5 + control, LV5‐CCAL1 + AMPK activator, LV5 + AMPK activator). Scale bars, 5 μm (*n* = 3). (C) qRT**–**PCR was performed to analyze the expression of Collagen Ⅰ, Sox9 and Collagen Ⅱ mRNAs in HC‐OA cells after different treatments (Blank, LV5‐CCAL1 + control, LV5 + control, LV5‐CCAL1 + AMPK activator, LV5 + AMPK activator). (D) WB was performed to analyze the expression of Collagen Ⅰ, Sox9, Collagen Ⅱ proteins in HC‐OA cells after different treatments (Blank, LV5‐CCAL1 + control, LV5 + control, LV5‐CCAL1 + AMPK activator, LV5 + AMPK activator). (E) The cell viability of HC‐OA cells after different treatments (Blank, LV5‐CCAL1 + control, LV5 + control, LV5‐CCAL1 + AMPK activator, LV5 + AMPK activator) were detected by CCK‐8 assay. (F) The proliferation capacity of HC‐OA cells after different treatments (Blank, LV5‐CCAL1 + control, LV5 + control, LV5‐CCAL1 + AMPK activator, LV5 + AMPK activator) were detected by clone formation assay. Data were analysed using Student’s *t*‐test and are expressed as the mean ± SD of three experiments. Symbols represent statistical significance (*, *P* < 0.05; **, *P* < 0.01).

In order to explore whether the AMPK signaling pathway is involved in the effect of CCAL1 on HC‐OA cells, the key factors involved in AMPK signaling pathway were detected after HC‐OA cells transfected with LV5‐CCAL1 and CCAL1 inhibitor. The results showed that p‐AMPK expression level in the LV5‐CCAL1 group was significantly lower, while the p‐AMPK expression level in the CCAL1 inhibitor group was higher than LV5 group, Blank group and inhibitor NC group (*P* < 0.05, Fig. 4A).

To further verify the effect of CCAL1 on HC‐OA cells by the AMPK signaling pathway, we added an activator of the AMPK signaling pathway to HC‐OA cells (Blank, LV5‐CCAL1 + activator control, LV5 + activator control, LV5‐CCAL1 + AMPK activator, LV5 + AMPK activator). The switching trend of the fibrosis‐prone phenotype of HC‐OA cells was significantly enhanced in the LV5‐CCAL1 + activator control group, compared with the other three groups, while significantly impeded in LV5 + AMPK activator group (Fig. 4B). After that, the expression levels of fibrotic genes and proteins were detected by qRT**–**PCR and WB. The results suggested that compared with the other three groups, Collagen I expression level significantly increased, while the expression level of Sox9 and Collagen II significantly decreased in the LV5‐CCAL1 + activator control group. The expression levels of Sox9 and Collagen II were significantly increased in LV5 + AMPK activator group, while the expression level of Collagen I significantly decreased (Fig. 4C,D). Besides, the cell viability was significantly increased in the LV5‐CCAL1 + activator control group, while significantly decreased in the LV5 + AMPK activator group (Fig. 4E). The results of colony formation assay demonstrated that the number of colonies in the LV5‐CCAL1 + activator control group (835 ± 6.56) was significantly increased, while significantly reduced in LV5 + AMPK activator group (204 ± 9.6), compared with the other three groups (*P* < 0.01, Fig. 4F). Therefore, CCAL1 could promote fibrosis, proliferation of HC‐OA cells by inhibiting the AMPK signaling pathway.

## Discussion

Osteoarthritis (OA) is a common chronic joint disease [8] and is characterized by the destruction of articular cartilage, sclerosis of subchondral bone, and inflammation of the synovium [11]. Aging is a primary risk factor for OA [12]. OA is prevalent in the elderly, and more than 10% of patients over the age of 60 worldwide [13]. The incidence of osteoarthritis is higher in women than in men [14]. Currently, most treatment methods focus on relieving the symptoms of OA, delay the remission of degeneration, and focus on pain relief and anti‐inflammation [15] and the mechanism of the onset and progression of OA has not been fully elaborated. Thus, in this study, we attempted to make contributions to the understanding of OA and novel therapy discovery.

CCAL1 served as a osteoprotegerin (OPG) encoding gene, belongs to the receptor‐associated factor (TNF) receptor superfamily [16]. OPG can inhibit bone resorption and maintain bone metabolic balance by binding to RANKL and blocking the binding of RANK and RANKL [17]. In addition, the OPG/RANKL/RANK ratio is an important indicator of bone health [18]. Ramos *et al*. [19] demonstrated that OPG was highly up‐regulated in OA cartilage. In this experiment, we found that CCAL1 was overexpressed in OA articular cartilage tissue and OA chondrocytes, and its high expression was positively correlated with the severity of OA. Meanwhile, CCAL1 could promote the fibrosis, proliferation and cell viability of HC‐OA cells *in vitro*.

AMPK is a serine/threonine‐protein kinase widely existed in eukaryotic cells [20]. Increasing evidence has revealed that AMPK can protect against fibrosis. Studies have shown that decreased AMPK activity in articular chondrocytes can lead to degenerative changes in articular cartilage and cause osteoarthritis [21]. Some researchers have demonstrated that AMPK agonists could delay the development of osteoarthritis *in vitro* [22]. Furthermore, the NF‐κB signaling pathway can mediate inflammatory fibrosis. Data have shown that most of the drugs used in the clinical treatment of OA have the effect of inhibiting the activity of NF‐κB [23]. Under the stimulation of external signals, the abnormal activation of NF‐κB leads to the expression of various genes, which is a key factor for OA development [24]. Activation of NF‐κB regulates the production of a large number of inflammatory mediators to lead to cartilage degeneration and accelerating the process of OA [25]. In this study, we demonstrated that CCAL1 could accelerate the process of OA by activating the NF‐κB signaling pathway and inhibiting the AMPK signaling pathway to promote fibrosis, proliferation, and increase cell viability of HC‐OA cells. In the future work, we will further verify and explore the development mechanism of osteoarthritis using primary cells and mouse models. In summary, we found that CCAL1 could promote the process of OA through NF‐κB/AMPK signaling pathway. The above research results provide a reliable basis for exploring the role of CCAL1 in OA and show a new direction for the clinical treatment of OA.

## Author contribution

HZ and HY conceived and designed the project, and wrote the paper. JM and HZ acquired the data. JZ and ZH analysed the data. YG modified the manuscript. All authors gave final approval of the version to be published, and agree to be accountable for all aspects of the work.

## Conflict of interest

The authors declare that they have no conflict of interest.

## Data Availability

The analyzed data sets generated during the present study are available from the corresponding author on reasonable request.
